# Biofilm-associated candidal thrombophlebitis

**DOI:** 10.1016/j.idcr.2023.e01733

**Published:** 2023-02-28

**Authors:** Shinnosuke Fukushima, Koichiro Yamamoto, Yasuhiro Nakano, Hideharu Hagiya, Fumio Otsuka

**Affiliations:** Department of General Medicine, Okayama University Graduate School of Medicine, Dentistry and Pharmaceutical Sciences, 2-5-1 Shikata-cho, Kitaku, Okayama 700-8558, Japan

**Keywords:** Biofilm, *Candida albicans*, Candidemia, Central-line associated bloodstream infection, Thrombophlebitis

## Case

**Fig. 1:**
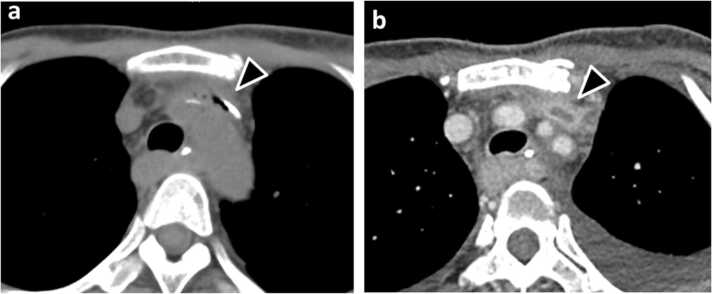
Computed tomography revealing candida thrombophlebitis in the brachiocephalic vein, a)Gas production surrounding a peripherally inserted central catheter **b)**Residual thrombus after catheter removal.

A 45-year-old Japanese woman receiving parenteral nutrition via a peripherally inserted central catheter (PICC) in the upper left arm was hospitalized with postoperative management for hypopharyngeal cancer. Blood culture detected *Candida albicans* and plain computed tomography (CT) revealed gas production surrounding the catheter in the brachiocephalic vein (Fig. 1a), leading to a diagnosis of central line-associated candidemia. The ophthalmological examination showed fungal endophthalmitis accompanying vitreous turbidity. We immediately extracted the PICC and administered 400 mg of intravenous fosfluconazole per day considering the antifungal susceptibility testing and eye penetration. Despite the antifungal treatment, candidemia persisted for 2 weeks, meanwhile four times of blood culture testings were repeatedly positive. A follow-up contrast-enhanced CT showed the disappearance of gas while a blood clot in the brachiocephalic vein remained (Fig. 1b). Considering the refractory condition due to candida biofilm-related thrombophlebitis, we additionally administered 200 mg of intravenous micafungin daily. Thereafter, recurrent candidemia promptly withdrew with a good clinical course.

Candidemia is a systemic mycosis caused by *Candida* species and commonly described as an opportunistic infection. Parenteral nutrition via a central venous catheter is a major risk factor of the disease [1]. The clinical course of candidemia is possibly complicated with a biofilm formation, and the mortality rate of biofilm-associated candidemia reported is extremely high at 70% [1]. Candida biofilm provides resistance to azole-class antifungals, wherein treatment with echinocandins or amphotericin B lipid formulation is reportedly promising [2], [3], [4]. The therapeutic course of the present case indicated that micafungin treatment was greatly effective in candidal thrombophlebitis, in spite of a treatment failure with fluconazole. In case candidemia persisted with thrombus formation, a candida biofilm targeted therapy may be required.

## Informed consent

Written informed consent was obtained from the patient to publish this case report.

## Ethical approval

This is case report, so we have informed consent.

## Funding

No authors have any funding in this case.

## Conflict of interest

No authors have any competing interests in this case.

## Author contribution

SF and YN contributed to patient care. SF wrote the manuscript, and KY and HH revised, and FO organized it.
